# Characteristics of Plant Community, Soil Physicochemical Properties, and Soil Fungal Community in a 22-Year Established Poaceae Mixed-Sown Grassland

**DOI:** 10.3390/jof11100756

**Published:** 2025-10-21

**Authors:** Pei Gao, Liangyu Lyu, Yunfei Xing, Jun Ma, Yan Liu, Zhijie Yang, Xin Wang, Jianjun Shi

**Affiliations:** 1 Academy of Animal Husbandry and Veterinary Sciences, Qinghai University, Xining 810016, China; y200954000466@qhu.edu.cn (P.G.); yb230909000074@qhu.edu.cn (L.L.); 2025066@qhmu.edu.cn (Y.X.); 13997471052@163.com (Y.L.); 15003600361@163.com (Z.Y.); 15110946395@163.com (X.W.); 2 Qinghai Province Key Laboratory of Adaptive Management of Alpine Grassland, Xining 810016, China; 3 State Key Laboratory of Ecology and Plateau Agriculture and Animal Husbandry in Sanjiangyuan Jointly Established by the Ministry of Provincial Affairs, Qinghai University, Xining 810016, China; 4 Graduate School, Qinghai Minzu University, Xining 810016, China; 5 Qinghai Grassland Improvement Experimental Station, Gonghe 813000, China; 18599055205@163.com

**Keywords:** Sanjiangyuan region, artificial grassland, grass Poaceae forage grass sowing combinations, soil microorganisms, soil physicochemical properties

## Abstract

This study aims to evaluate the restoration effect of artificially mixed-sown grasslands by investigating the characteristics of plant communities and soil fungal communities in long-term (22-year-established) artificial grasslands under six Poaceae mixture combinations. The experiment took mixed-sown grasslands of grass species established in 2002 on the Qinghai–Tibet Plateau as the research object. It employed ITS gene high-throughput sequencing technology to construct a fungal community distribution map and combined it with FUNGuild (Functional Guilds of Fungi) functional predictions to analyze fungal species abundance, structural diversity, molecular co-occurrence networks, and functional characteristics. By integrating Mantel test and RDA (redundancy analysis), we identified key environmental factors driving soil microbial community structure in mixed-sown grasslands and revealed the plant–soil–microbe interaction mechanisms in a Poaceae mixture grassland. The results showed that the HC treatment (a mixture of three grass species) significantly enhanced plant biomass and soil nutrient accumulation. In 2023 and 2024, its aboveground biomass increased by 66.14% and 60.91%, respectively, compared to the HA treatment (monoculture). Soil organic matter increased by 52.32% and 48.35%, while electrical conductivity decreased by 48.99% and 51.72%, respectively. The fungal community structure improved under the HD treatment (a mixture of four grass species), with an increased abundance of the dominant phylum Ascomycota and a 14.44% rise in the Shannon index compared to the HA treatment. The network complexity under the HF treatment (a mixture of six grass species) increased (with edge numbers reaching 494), while the functional abundance of plant pathogen was significantly lower than that under the HA treatment. Mantel test and RDA revealed that SEC (soil electrical conductivity) was significantly positively correlated with pH, while both exhibited negative correlations with other plant and soil physicochemical indicators. Moreover, SEC emerged as the core factor driving fungal community assembly. Mixed sowing of three to four grass species effectively regulated soil electrical conductivity, simultaneously enhancing plant biomass, soil nutrients, and fungal community diversity, representing an optimal strategy for artificial restoration of degraded grasslands.

## 1. Introduction

The alpine meadow ecosystem, with cold-tolerant herbaceous plants as its dominant species, possesses characteristics such as resistance to low temperatures, low oxygen levels, and strong radiation. It not only performs crucial ecological service functions, including water conservation and carbon sink regulation, but also holds economic value by supporting the development of animal husbandry. Thus, it serves as an important vehicle for the coordinated development of ecological conservation and sustainable utilization [1]. However, affected by multiple factors such as intensified climate warming and aridification, overgrazing, and outbreaks of rodent populations like plateau pikas, ecological degradation in this ecosystem has become increasingly prominent. This is manifested by a significant reduction in vegetation coverage, a continuous decline in species richness, deterioration of topsoil fertility, and weakened functions of soil fungal communities, ultimately leading to patchy degradation characteristics in the alpine meadow ecosystem. Consequently, both its ecological barrier effectiveness and forage production capacity have markedly declined [2,3]. To address this severe situation, the local government has implemented a set of comprehensive restoration measures targeting degraded alpine meadows, including artificial grassland establishment [4], nutrient supplementation [5], rodent pest control [5], and fencing for natural regeneration [6]. Among these, artificial grassland establishment stands out as a key technical approach. By optimizing grassland community structure, improving soil physicochemical properties, and enhancing soil fungal community diversity, ecosystem recovery and functional enhancement are effectively promoted, serving as crucial initiatives for improving regional ecological conditions and ensuring ecological security [4].

The establishment of artificial grasslands can significantly enhance plant community coverage, height, biomass, and species diversity in the short term, while effectively increasing soil nutrient content and optimizing the structure and function of soil fungal communities [4,7]. According to research by scholars such as Tian et al. [7], establishing short-term artificial grasslands through monoculture sowing in the Loess Plateau region can significantly enhance grassland carbon sequestration rates and accelerate the carbon cycling process in ecosystems. Scholars such as Hou et al. [4] confirmed that establishing artificial grasslands through monoculture sowing of grasses on the Qinghai–Tibet Plateau can effectively increase vegetation coverage and enhance forage yield. However, in production practice, it has been found that artificial grasslands established through monoculture sowing of grasses suffer from issues such as low community diversity, soil fertility degradation, and nutrient imbalance. These problems subsequently lead to reduced diversity and functional decline in soil fungal communities, undermining ecosystem stability and hindering the sustainable development of alpine grasslands [8,9]. In contrast, Poaceae mixture sowing technology achieves the dual goals of soil improvement and vegetation restoration simultaneously by optimizing the allocation of plant functional groups [10]. The study by Guo et al. [11] demonstrated that mix sowing forage could significantly reduce soil erosion, improve forage nutritional composition, and enhance grassland productivity and community stability. Further research by Robinson et al. [12] confirmed that mixed-sown communities could boost productivity through interspecific complementary effects and, in some cases, suppress the expansion of invasive species while promoting the natural restoration of native vegetation, offering a more sustainable technical approach for ecological restoration of alpine meadows.

Current research predominantly focuses on evaluating the short-term restoration effects (3–5 years) of artificial grasslands [4,7,8,9], while systematic studies on grass mixtures sown over 20 years in high-altitude regions remain scarce. In particular, the coupling mechanisms among soil fungal community structure, soil physicochemical properties, and vegetation restoration require further in-depth analysis. This study takes a 22-year-established Poaceae mixed-sown grassland on the Qinghai–Tibet Plateau as the research object, systematically exploring the variation patterns of plant community characteristics, soil physicochemical properties, and soil fungal community structure under different Poaceae mixture sowing treatments. It focuses on two key scientific questions: First, how do different Poaceae mixture sowing treatments drive the differential evolution of soil fungal community composition and functional traits? Second, under the context of long-term restoration (22 years), how is the interaction mechanism between fungal community structure and soil–vegetation factors established in Poaceae mixed-sown grasslands? The research results will offer critical theoretical support and technical guidance for the ecological restoration practices of degraded alpine meadows and the sustainable management of mixed-sown Poaceae grass. This will contribute to achieving the dual objectives of enhancing ecosystem functions and ensuring regional ecological security.

## 2. Materials and Methods

### 2.1. Overview of the Study Area

**Figure 1:**
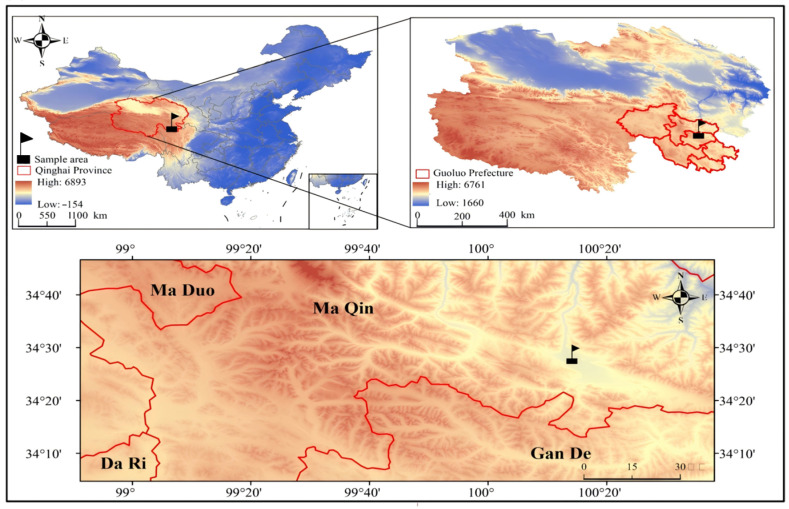
Geographical location of the experimental area. Note: This figure is based on the standard map, Approval No.: GS (Beijing, China) No. 1061 (2022), and the data were obtained from the Standard Map Service website of the National Administration of Surveying, Mapping and Geoinformation.

The study area is located in Maqin County, Guoluo Tibetan Autonomous Prefecture, Qinghai Province, China (32°31′ N to 35°40′ N, 97°54′ E to 101°50′ E), at an elevation of 3764 m (Figure 1). The region exhibits typical plateau continental climate characteristics, with an annual mean temperature of −3.5 °C, annual precipitation ranging from 423 mm to 565 mm, and annual sunshine duration of 2313 to 2607 h [13]. There is no absolute frost-free period. The healthy grasslands in this area are predominantly alpine Kobresia meadows, with the soil type classified as alpine meadow soil. The plant community is dominated by the constructive species *Carex alatauensis*, accompanied by cold-tolerant species such as *Carex parvula* and *Elymus nutans*. The study area has suffered from severe degradation, forming secondary bare land characterized by a “rodent damage–overgrazing” pattern. This degradation is primarily caused by burrowing, mound-building, and herbivory by rodents such as plateau pikas and plateau zokors, as well as overgrazing. Prior to the establishment of artificial grasslands, toxic weeds including *Potentilla anserina* and *Ligularia virgaurea* served as the dominant constructive species, with bare patch coverage reaching or exceeding 80% and the proportion of edible forage being 5% or less.

### 2.2. Experimental Design

#### 2.2.1. Mixed-Sowing Combinations

In this experiment, six grass species suitable for growth in alpine regions were selected as experimental materials. Specifically, they include *Elymus nutans* Griseb., *Festuca sinensis* cv. Qinghai, *Festuca kryloviana* cv. Huanhu, *Elymus breviaristatus* F. cv. Qinghai, *Poa crymophila* cv. Qinghai and *Poa poophagorum*. The purity of seeds for all tested grass species is ≥92%, and their germination vigor is ≥85%. The experiment established six sowing treatments: The control treatment HA consisted solely of *Elymus nutans* Griseb. The experimental treatments HB, HC, HD, HE, and HF were mixed-sowing combinations of two, three, four, five, and six grass species, respectively. The specific combinations were as follows: HB(*Elymus nutans* Griseb. + *Poa crymophila* cv. Qinghai), HC(*Elymus nutans* Griseb. + *Poa crymophila* cv. Qinghai + *Festuca sinensis* cv. Qinghai), HD(*Elymus nutans* Griseb. + *Poa crymophila* cv. Qinghai + *Festuca sinensis* cv. Qinghai + *Poa poophagorum*), HE(*Elymus nutans* Griseb. + *Poa crymophila* cv. Qinghai + *Festuca sinensis* cv. Qinghai + *Poa poophagorum* + *Festuca kryloviana* cv. Huanhu), HF(*Elymus nutans* Griseb. + *Poa crymophila* cv. Qinghai + *Festuca sinensis* cv. Qinghai + *Poa poophagorum* + *Festuca kryloviana* cv. Huanhu + *Elymus breviaristatus* F. cv. Qinghai). All mixed-sowing treatments employed equal sowing proportions.

#### 2.2.2. Experimental Plot Design and Seeding Quantity per Plot

This study employed a completely randomized block design, dividing the experimental site into three blocks. Each block contained six experimental plots (3 m × 4 m), corresponding to the six sowing treatments (HA, HB, HC, HD, HE, and HF). The specific randomization process was as follows: Within each block, HA (the monoculture control of *Elymus nutans*) and HB to HF (the mixed-sowing treatments) were randomly assigned to the six plots using a random number generator, ensuring that each treatment appeared only once and in a random position within each block. The control treatment HA was present in every block, but its specific location was determined through randomization to avoid positional bias. A 1.0 m buffer strip was established between adjacent plots to minimize edge effects, and a 2.0 m isolation strip was set up between blocks. The site preparation involved deep tillage (25 cm plowing depth), followed by disk harrowing (soil particle size after crushing <2 cm), and leveling the bed surface with a heavy roller. Sowing was conducted using manual row sowing, with a sowing depth of 2.0–3.0 cm and row spacing of 20 cm. For base fertilizer application, 150 kg·hm^−2^ of diammonium phosphate (DAP) compound fertilizer was used, supplemented with 75 kg·hm^−2^ of urea. The seeding rates for the six grass species were as follows: *Elymus nutans* Griseb. at 3.0 g·m^−2^, *Elymus breviaristatus* F.cv. ‘Qinghai’ at 3.0 g·m^−2^, *Poa pratensis* L. ‘Qinghai’ at 0.75 g·m^−2^, *Poa crymophila* cv. ‘Qinghai’ at 0.75 g·m^−2^, *Festuca kryloviana* cv.Huanhu at 0.75 g·m^−2^, and *Festuca sinensis* cv. ‘Qinghai’ at 2.25 g·m^−2^. The seeding rate for each experimental plot was determined according to Formula (1):

(seeding rate of single grass species)/n,(1)

where n = number of species in the combination.

#### 2.2.3. Field Management

Systematic rodent control measures were implemented in the experimental site (covering core area + 200 m buffer zone). Rodent damage prevention and control were initiated in March annually according to phenological patterns, employing bait–physical coordinated control techniques to maintain rodent density below the damage threshold. Seasonal rotational grazing management was applied to experimental plots: grazing was prohibited from April to November, and grazing was allowed from December to March of the following year.

### 2.3. Plant Community Survey and Soil Sample Collection

#### 2.3.1. Investigation of Plant Community

In August 2023 and the corresponding period of 2024, five quadrats (0.5 m × 0.5 m each) were randomly set up in each of the 18 experimental plots to conduct vegetation surveys in the sample plots, with a total of 90 quadrats surveyed. The sward height was measured using a tape measure (with an accuracy of ±1 mm), with nine measurements taken per quadrat. The community coverage was determined using the point-quadrat method, with one measurement per quadrat. The community density was assessed by the actual counting method, recording the number of plant individuals within each quadrat, with one measurement per quadrat. Plants within the quadrats were cut at ground level. The fresh plant samples were placed in an oven for enzyme deactivation (105 °C for 30 min) and then dried at a constant temperature of 65 °C until a constant weight was achieved. The dry weight was measured using an electronic balance (accuracy: 0.0001 g) as the aboveground biomass.

#### 2.3.2. Collection of Soil Samples

In the 90 quadrats across 8 experimental plots, a soil auger with an inner diameter of 5 cm, in combination with the five-point sampling method, was employed to collect original soil samples from the 0–10 cm soil layer. Five original soil samples were obtained from each quadrat, resulting in a cumulative total of 450 original soil samples. After pre-treatment involving root screening and gravel removal, the original soil samples from each experimental plot were thoroughly mixed to prepare measured soil samples, yielding a total of 18 measured soil samples [14]. These measured soil samples were divided into three groups according to experimental requirements: The first group was air-dried and used for the determination of soil nutrient indicators such as soil organic matter (SOM), total nitrogen (TN), and total phosphorus (TP); the second group was refrigerated at −20 °C for the detection of soil gravimetric water content(SWC), pH value, and electrical conductivity(SEC); the third group was stored in a −80 °C ultra-low-temperature freezer specifically for high-throughput sequencing analysis of soil fungi [14].

### 2.4. Determination of Soil Indicators

#### 2.4.1. Determination of Soil Physical and Chemical Properties

Soil physicochemical properties including soil electrical conductivity (SEC), soil water content (SWC), pH value, soil organic matter (SOM), total nitrogen (TN), and total phosphorus (TP) were determined according to Soil Agrochemical Analysis (3rd Edition) [15].

#### 2.4.2. Soil Fungal DNA Extraction, PCR Amplification, and High-Throughput Sequencing

Soil fungal DNA extraction, PCR amplification, and high-throughput sequencing were commissioned to Shanghai Majorbio Biotechnology Co., Ltd. The workflow commenced with DNA extraction and quality control, where agarose gel electrophoresis (1.0%) and NanoDrop 2000 spectrophotometry (Thermo Scientific, Waltham, MA, USA) were employed for quality assessment and quantification of environmental total DNA. After assessing nucleic acid purity and concentration and ensuring sample integrity, qualified DNA was stored at −80 °C for ultra-low-temperature preservation. Subsequent PCR amplification was performed using primer pair ITS1F (5′-CTTGGTCATTTAGAGGAAGTAA-3′) and ITS2R (5′-GCTGCGTTCTTCATCGATGC-3′) targeting fungal gene sequences. The 25 μL reaction system contained the following: 10 ng template DNA, 0.2 μM primers, 1 × PCR buffer, 0.2 mM dNTPs, 1.5 mM MgCl_2_, and 1 U Taq DNA polymerase. The amplification protocol was set as follows: initial denaturation at 94 °C for 5 min; 35 cycles of denaturation at 94 °C for 30 s, annealing at 55 °C for 30 s, and extension at 72 °C for 45 s; followed by a final extension at 72 °C for 10 min. Amplified products were screened for target fragments via 2% agarose gel electrophoresis and purified using a PCR purification kit to remove impurities. The purified product is quantitatively standardized by using the Qubit 4.0 fluorescence quantifier (Thermo Fisher Scientific, Waltham, MA, USA) to ensure uniform concentration. In the high-throughput sequencing stage, the purified amplicon was double-ended sequenced by the Illumina PE300 platform. The original sequencing data were filtered by fastp software (v0.19.6) for quality control (including removing low-quality sequences, linker contamination, and primer residues), and then the sequences were spliced by FLASH (v1.2.7) to generate high-quality non-chimeric sequences.

### 2.5. Data Analysis

The raw data were organized using Microsoft Excel 2019 software, and one-way analysis of variance (ANOVA) was conducted using SPSS 27.0. We used QIIME2 software (2024.10) to generate rarefaction curves, species relative abundance plots, PCoA plots (based on Bray–Curtis dissimilarity), and phylogenetic heatmaps, as well as to calculate α diversity indices (including OTUs, Shannon index, Simpson index, Pielou index, Chao1 index, and ACE index). To further explore the differences in community structure among grouped samples, the LEfSe statistical analysis method was selected to conduct significance tests for the differences in species composition and community structure of the grouped samples. By associating the annotation results of amplicons with corresponding functional databases, the FUNGuild software can be selected to perform functional prediction analysis on the microbial communities in ecological samples. The single-factor molecular network diagram is constructed using Networkx (version 1.11), and interspecific associations are calculated. All visual analyses of soil fungi are carried out on the Majorbio Bioinformatics Cloud Platform (https://www.majorbio.com), URL (accessed on 20 May 2025). Heatmaps were constructed using R 3.5.2 software based on Pearson correlation coefficients to illustrate the relationships among plant community characteristics, soil physicochemical properties, fungal community diversity, and fungal community abundances at the phylum and genus levels. Redundancy analysis (RDA) was performed using Canoco 5.0 software to quantify the explanatory rates of plant community characteristics and soil physicochemical factors on fungal community diversity and fungal community abundances at the phylum and genus levels.

## 3. Results

### 3.1. Effects of Poaceae Mixture Sowing on Vegetation Community Characteristics

Significant differences were observed in vegetation height, coverage, density, and aboveground biomass among different Poaceae mixture sowing treatments. Specifically, the HC and HD treatments demonstrated significantly higher values for the aforementioned vegetation community characteristics compared to the monoculture control (HA) treatment (Table 1). After 22 years of planting (2023), the vegetation height of six treatments ranged from 32.51 cm to 45.86 cm. Among them, the HD treatment exhibited the highest vegetation height (45.86 cm), showing a significant increase of 28.14% compared to the HA treatment (*p* < 0.05). Similarly, vegetation coverage under the HD treatment reached 92.33%, representing a significant improvement of 28.24% over the HA treatment (*p* < 0.05). The HC treatment demonstrated the highest density (913.33 plants·m^−2^) and aboveground biomass (1580.00 g·m^−2^), with significant increases of 29.00% and 66.14% compared to the HA treatment, respectively (*p* < 0.05).

In the 23rd year of planting (2024), the trends in vegetation height, coverage, density, and aboveground biomass remained consistent with those observed in 2023. The HC treatment maintained the highest vegetation height (50.00 cm) and aboveground biomass (1645.00 g·m^−2^), showing significant increases of 40.63% and 52.36% over the HA treatment, respectively (*p* < 0.05). Among mixed-sowing treatments, vegetation coverage ranged from 77.00% to 95.22%, and density ranged from 665.33 plants·m^−2^ to 942.33 plants·m^−2^, representing increments of 2.22% to 26.55% in coverage and 4.11% to 41.63% in density compared to the unicast HA treatment. In summary, the HC treatment performed best in optimizing vegetation community structure (particularly density and aboveground biomass), while the HD treatment showed significant advantages in height and coverage. The HA treatment exhibited the poorest performance.

### 3.2. Impact of Poaceae Mixture Sowing on Soil Physicochemical Properties

**Figure 2:**
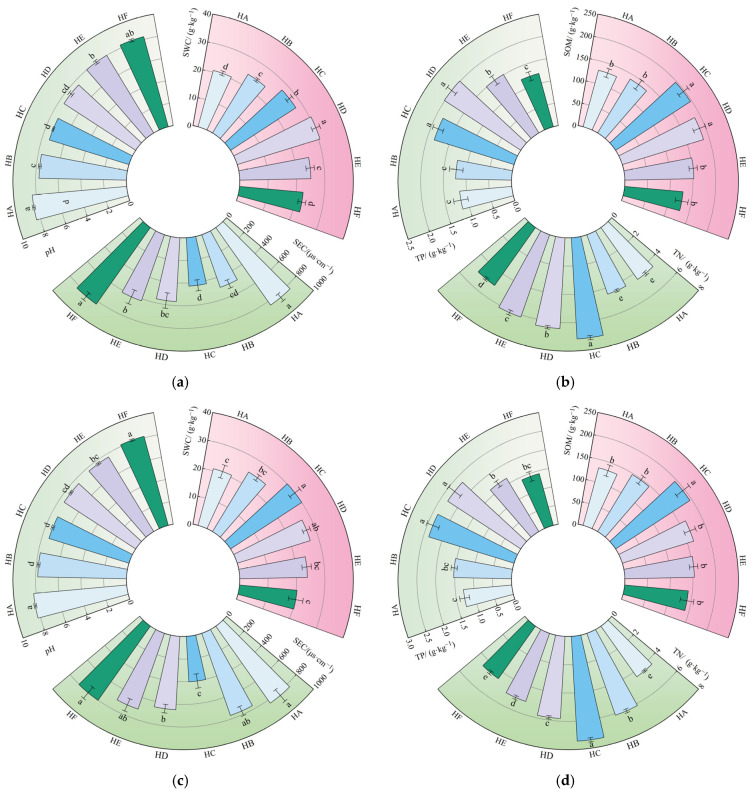
Changes in soil physical and chemical properties under different mixed-sowing treatments. Note: (**a**) index measured after 22 years of planting: pH, SWC, and SEC; (**b**) index measured after 22 years of planting: TP, SOM, and TN; (**c**) index measured after 22 years of planting: pH, SWC, and SEC; (**d**) index measured after 22 years of planting: TP, SOM, and TN. Different lowercase letters in the same treatment group showed significant differences at the level of *p* < 0.05.

Different Poaceae mixture sowing treatments significantly altered soil physicochemical properties, specifically manifesting as significant differences in soil water content (SWC), electrical conductivity (SEC), pH, soil organic matter (SOM), total nitrogen (TN), and total phosphorus (TP) contents among the treatments. Among them, compared with the monoculture control (HA) treatment, the HD and HC treatments exhibited significantly increased nutrient contents such as SOM, TN, and TP, along with a significant decrease in SEC (Figure 2). After 22 years of planting (2023), soil water content across the six treatments ranged from 20.62% to 30.49%. Notably, the HD treatment exhibited the highest SWC (30.49%), showing a significant increase of 47.87% compared to the HA treatment (*p* < 0.05). SEC and pH were the lowest in HC treatment, at 427.67 μs·cm^−1^ and 7.34, respectively, which were 48.99% and 12.10% lower than those in HA treatment, with significant difference (*p* < 0.05). The contents of SOM (197.00 g·kg^−1^) and TN (7.16 g·kg^−1^) were the highest in HC treatment, and were 52.32% and 59.47% higher than those in HA treatment, respectively, with significant differences (*p* < 0.05). In the five mixed-sowing treatments, the TP content ranged from 1.22 g·kg^−1^ to 2.01 g·kg^−1^, which was 6.09% to 74.78% higher than that of the unicast HA treatment.

After 23 years of planting (2024), the change trend of soil physical and chemical indexes is basically the same as that in 2023. SWC was the highest in HC treatment (29.99%) and the lowest in HF treatment (20.33), with significant difference (*p* < 0.05). In the mixed seeding treatment, the SEC was between 401.67 μs·cm^−1^ and 802.33 μs·cm^−1^, and the pH was between 7.38 and 8.13, which were 3.57–107.14% and 1.11–10.22% lower than that in the unicast HA treatment, respectively. The contents of SOM, TN, and TP in HC treatment were the highest, at 195.33 g·kg^−1^, 57.41 g·kg^−1^ and 2.36 g·kg^−1^, respectively, which were significantly higher than those in HA treatment by 48.35%, 69.18%, and 84.38%(*p* < 0.05). To sum up, HC treatment has the best effect on improving soil physical and chemical properties, followed by HD treatment, and HA treatment has the worst physical and chemical properties.

### 3.3. Effect of Species Composition of Soil Fungi Community Mixed Sowing with Poaceae Forage Grass

#### 3.3.1. ITS Sequencing Results and OUTs Quantity Changes in Soil Fungi

**Figure 3:**
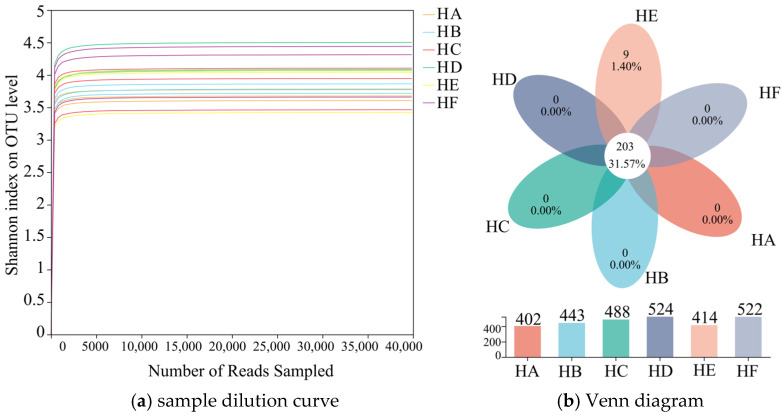
Dilution curve and Venn analysis of fungal community in soil samples. Note: (**a**) dilution curves, (**b**) Venn diagrams.

When the number of reads sampled reached 5000, the Shannon index dilution curve at the OTU level reached saturation and stabilized, indicating that the sequencing depth had adequately covered the fungal community in the samples and that the sequencing data were reliable (Figure 3a). Venn analysis revealed that a total of 643 OTUs were detected across the six Poaceae mixture sowing treatments (Figure 3b). Among these, the HD treatment exhibited the highest number of OTUs (524), while the HA and HE treatments showed the lowest, with 404 and 414 OTUs, respectively. The six treatments shared 203 common OTUs, accounting for 31.57% of the total OTUs. Unique OTUs were predominantly enriched in the HE treatment, with nine unique OTUs, representing 1.40% of the total.

#### 3.3.2. Changes in Soil Fungal Community Composition and Relative Abundance

**Figure 4:**
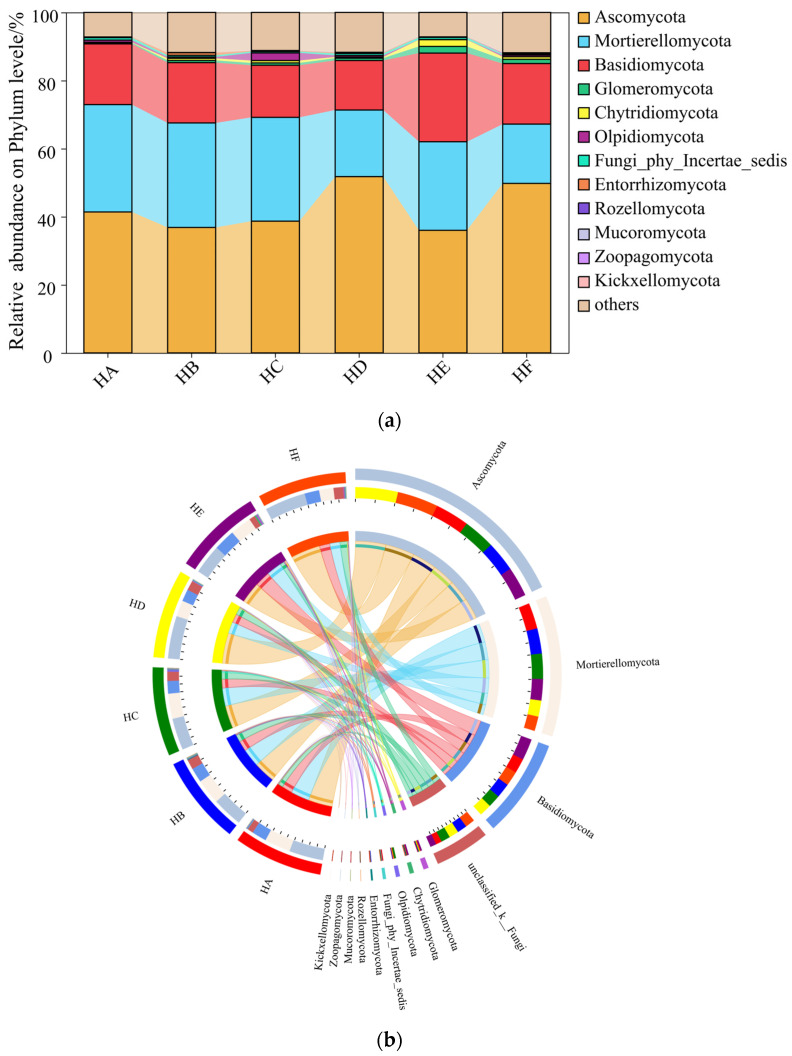
Changes in relative abundance of fungi in soil samples at the level of phylum. Note: (**a**) Circos, (**b**) CommunityBarPie.

The abundances of the top 12 fungal phyla underwent significant changes in the soils of the six Poaceae mixture sowing treatments (Figure 4). Ascomycota (36.00~51.78%) was the dominant phylum, followed by Mortierellomycota (17.42~31.53%) and Basidiomycota (14.54~26.04%). Significant differences in soil fungal abundance were observed among treatments. Specifically, the HD treatment exhibited a significantly higher relative abundance of Ascomycota compared to other treatment groups; however, the abundances of Mortierellomycota and Basidiomycota in the HD treatment were significantly lower than those in other treatments.

**Figure 5:**
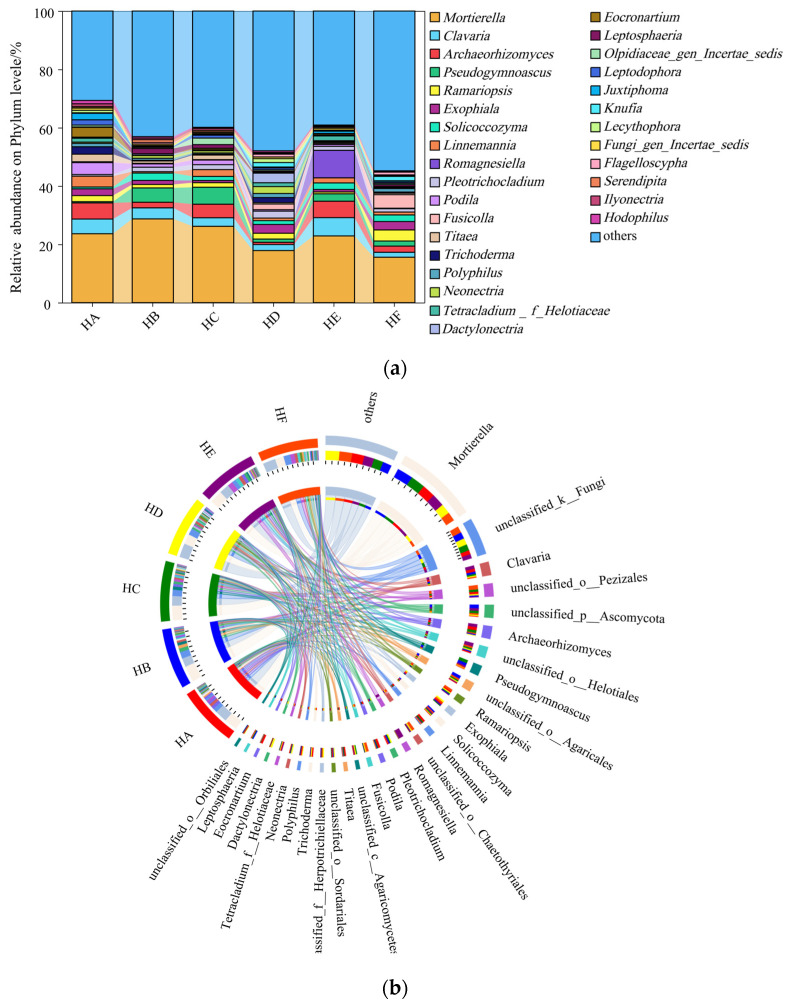
Variation in relative abundance of fungi community in soil samples at generic level. Note: (**a**) Circos, (**b**) CommunityBarPie.

The abundances of the top 30 fungal genera experienced significant variations in the soils of the six Poaceae mixture sowing treatments (Figure 5). Among the six mixed-sowing treatments of Poaceae forage grass, the top eight fungal genera with higher relative abundance were identified (Figure 5): *Mortierella* (15.58~28.77%), *Clavaria* (1.69~6.28%), *Archaeorhizomyces* (0.77~5.63%), *Pseudogymnoascus* (0.42~5.8%), *Ramariopsis* (0.65~3.80%), *Exophiala* (0.75~3.00%), *Solicoccozyma* (0.63~2.53%), and *Linnemannia* (0.48~3.72%). Notably, *Romagnesiella* (0.00~9.43%) was exclusively detected in the HE treatment, reaching a remarkable abundance of 9.43%. Further analysis revealed significant differences in genus-level soil fungal abundance among the mixed-sowing treatments of Poaceae forage grass. Specifically, the relative abundances of *Ramariopsis* and *Exophiala* in the HD and HF treatments were significantly higher than those in other treatments. Conversely, the relative abundances of *Mortierella* and *Clavaria* in the HD and HF treatments were significantly lower compared to other treatments.

#### 3.3.3. LEfSe Analysis of Soil Fungal Community

**Figure 6:**
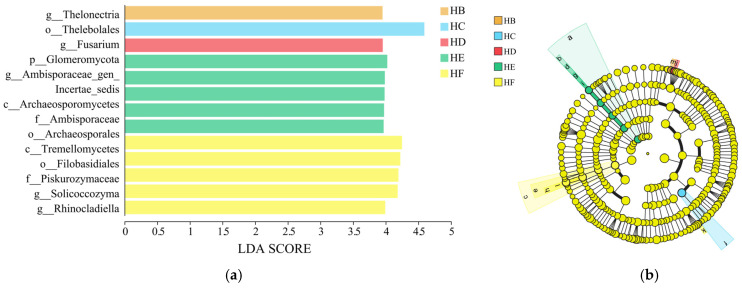
LEfSe analysis of fungal community in soil samples. Note: (**a**) LEfSe analysis (LDA > 4.0), (**b**) LDA value of indicator species (LDA > 4.0).

LEfSe analysis revealed distinct biomarkers in soil fungal communities among the six mixed-sowing treatments of Poaceae forage grass (Figure 6). Using an LDA threshold > 3.5 as the criterion, a total of 13 statistically significant biomarkers were identified (HB = 1, HC = 1, HD = 1, HE = 5, and HF = 5). At the taxonomic level, the highest-scoring biomarkers for HB, HC, HD, HE, and HF treatments were genus g__*Thelonectria*), o__*Thelebolales*, g__*Fusarium*, p__*Glomeromycota*, and c__*Tremellomycetes*, respectively. Notably, o__*Thelebolales* in the HC treatment achieved the highest score (LDA threshold > 4.5), exerting a significant influence on the fungal community structure.

### 3.4. Effect of Soil Fungal Community Diversity of Soil Fungi Community Mixed Sowing with Poaceae Forage Grass

#### 3.4.1. Changes Inf α Diversity of Soil Fungal Community

**Figure 7:**
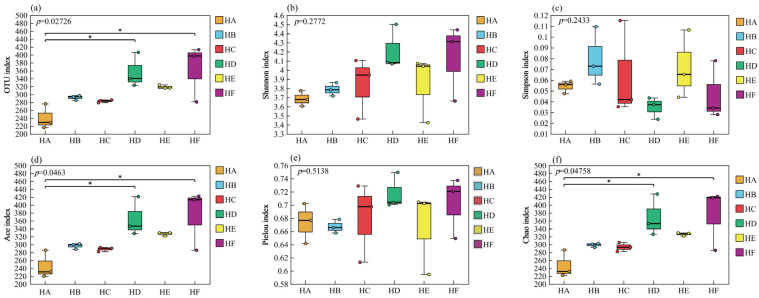
Difference in α diversity of fungal communities in soil samples. Note: (**a**) number of OTU, (**b**) Shannon index, (**c**) Simpson index, (**d**) Ace index, (**e**) Pielou index, (**f**) Chao1 index. * represents *p* < 0.05.

Different Poaceae mixture sowing treatments of grass species significantly regulated the α diversity of soil fungal communities. Among them, the OTU number, Shannon index, Ace index, Pielou index, and Chao1 index in the HD and HF treatments were significantly higher than those in the monoculture control (HA) treatment (Figure 7). The Shannon index (4.22) and Pielou index (0.72) of HD treatment were the highest, which were 14.44% and 6.62% higher than those of HA treatment, respectively. The number of OTU (363.67), Ace index (373.58), and Chao1 index (375.14) in HF treatment were the highest, which were 51.32%, 52.26%, and 52.19% higher than those in HA treatment, followed by HD treatment, both of which were significantly higher than those in HA treatment (*p* < 0.05). Simpson index of the six treatment groups was HB > HE > HC > HA > HF > HD, and the Simpson index of HD treatment was the lowest, only 0.029.

#### 3.4.2. Changes in β Diversity (PCoA) of Soil Fungal Community

**Figure 8:**
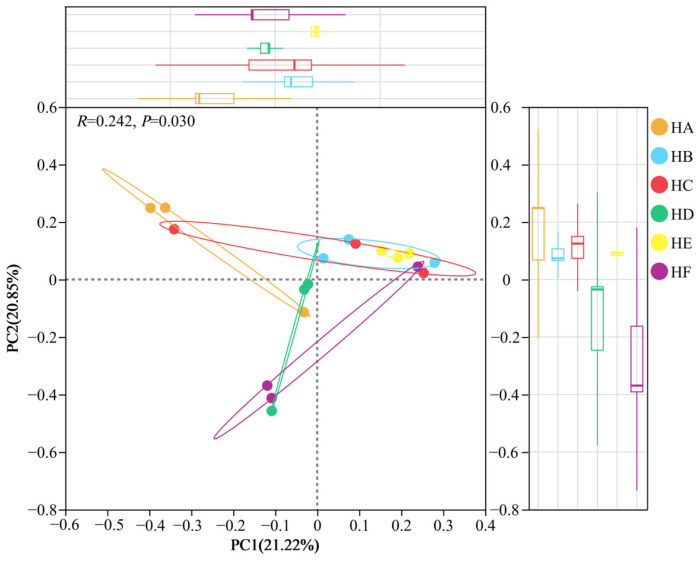
PCoA analysis of fungal community in soil samples.

The β-diversity analysis (PCoA) of soil fungal communities based on Bray–Curtis distance revealed significant differences in β-diversity among different treatments (*p* = 0.03) (Figure 8). The HE treatment exhibited the highest within-group homogeneity, with sample points showing tightly clustered characteristics, while the HA, HC, and HF treatments demonstrated lower within-group homogeneity. The first two principal coordinate axes (PC1 and PC2) cumulatively explained 42.07% of the variance in sample composition, with PC1 accounting for 21.22% and PC2 for 20.85%.

### 3.5. The Impact of Poaceae Mixture Sowing on the Single-Factor Molecular Network of Soil Fungal Communities

**Figure 9:**
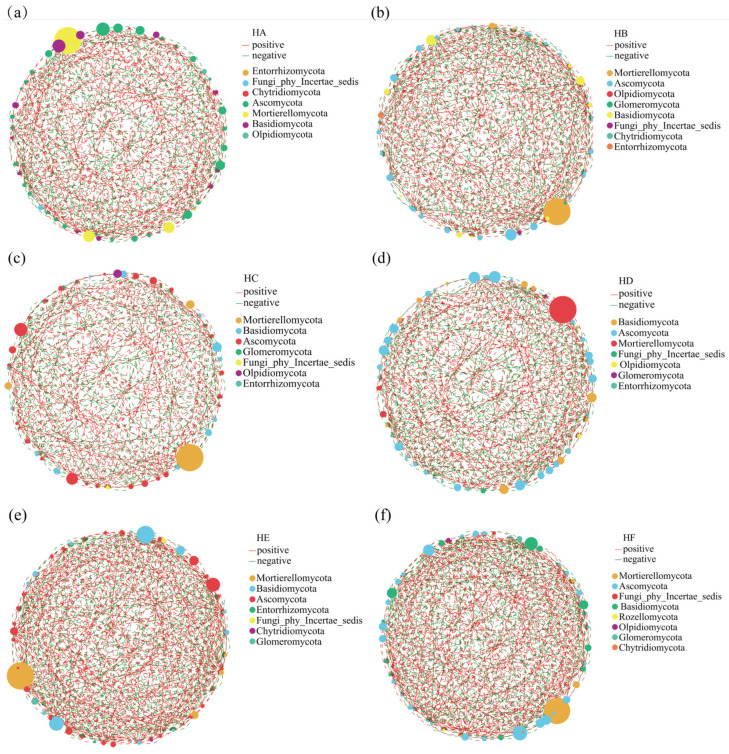
Single-factor molecular network diagram of fungal community in soil samples. Note: node color and size indicate species type and importance; line color indicates positive or negative correlation; and red positive, green negative, and the number of lines indicate whether the species are closely related. (**a**) HA, (**b**) HB, (**c**) HC, (**d**) HD, (**e**) HE, (**f**) HF.

Different Poaceae mixture sowing treatments significantly reshaped the univariate molecular networks of soil fungal communities, specifically manifesting as significant differences in the number of network edges, nodes, and positive correlations among the treatments (Figure 9). Research indicates that the topological characteristics of soil fungal communities exhibit significant differences among different treatments. Among these, the HB (401 edges), HE (490 edges), and HF (494 edges) treatments exhibited the highest network edge numbers, with increases of 8.97%, 33.15%, and 34.24% compared to the HA treatment (368 edges), respectively. The number of nodes showed insignificant variation among different mixed-sowing treatments of Poaceae forage grass, all ranging between 48 and 50. Specifically, the HC, HE, and HF treatments had the maximum node numbers, all at 50. For all treatments, the proportion of positive correlation edges in the molecular networks exceeded 50% (HA: 56.52%; HB: 54.49%; HC: 54.66%; HD: 51.79%; HE: 53.47%; HF: 54.25%). In the associated networks of soil fungal communities, the key connecting nodes (critical hubs) shifted with changes in the mixed-sowing combinations. The core hubs of the HA treatment were Entorrhizomycota, Fungi_phy_Incertae_sedis, and Chytridiomycota, while the HB, HC, HD, HE, and HF treatments were dominated by Mortierellomycota, Ascomycota, Olpidiomycota, and Basidiomycota as the leading hubs.

### 3.6. Effect of Poaceae Mixture Sowing on Soil Fungal Community Function

**Figure 10:**
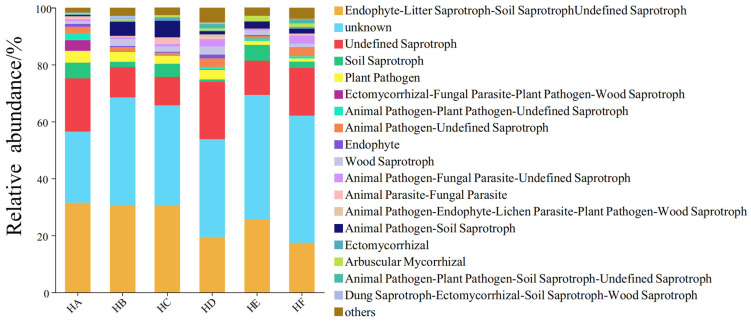
Functional classification diagram of FUNGuild fungi community in soil samples.

Based on the FUNGuild functional prediction, a total of 18 fungal trophic modes were identified across the six mixed-sowing treatments of Poaceae forage grass (Figure 10). The top five trophic modes in terms of functional abundance proportion were as follows: Endophyte-Litter Saprotroph-Soil Saprotroph-Undefined Saprotroph (17.41–31.48%), Unknown (25.00–44.65%), Undefined Saprotroph (9.90–20.22%), Soil Saprotroph (0.78–5.64%), and Plant Pathogen (1.04–4.13%). Specifically, the HA treatment was dominated by the Endophyte-Litter Saprotroph-Soil Saprotroph-Undefined Saprotroph, while the HB, HC, HD, HE, and HF treatments were primarily dominated by the Unknown functional group. The abundance of the unknown functional group in the HA treatment was significantly lower than that in the other treatments. Additionally, the HF treatment exhibited a significantly lower abundance of plant pathogen.

### 3.7. Soil Fungal Community Clustering Heatmap

**Figure 11:**
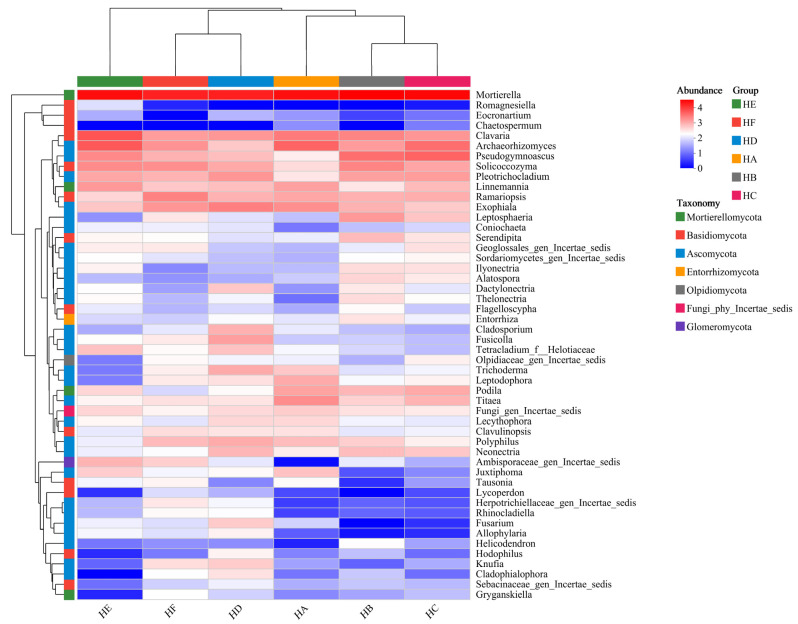
Soil sample fungal community clustering heatmap.

Phylogenetic heatmap analysis and cluster analysis based on the genus-level fungal community structure revealed that the six mixed-sowing treatments of Poaceae forage grass exerted significant regulatory effects on soil fungal composition, with the top 50 dominant fungal genera by relative abundance exhibiting significant specificity across different treatments (Figure 11). The HA treatment specifically clustered *Mortierella*, *Clavaria*, *Archaeorhizomyces*, and *Titaea*. The HB treatment was dominated by *Mortierella*, *Pseudogymnoascus*, and *Solicoccozyma*. The HC treatment showed significant enrichment of *Mortierella*, *Archaeorhizomyces*, and *Pseudogymnoascus*. The HD treatment was characterized by the enrichment of *Mortierella*, *Exophiala*, *Pleotrichocladium*, and *Fusicolla*. The HE treatment was core-enriched with *Mortierella*, *Clavaria*, and *Archaeorhizomyces*. The HF treatment was primarily dominated by *Mortierella*, *Ramariopsis*, and *Exophiala*. *Mortierella*, as a key hub genus, dominated all treatments, while the specific enrichment of *Exophiala* in HD and HF treatments was potentially linked to its lignin-degrading function. Based on the composition of soil fungi, the six mixed-sowing treatments of Poaceae forage grasses can be divided into two groups. Group 1 consists of the HE treatment, while Group 2 comprises the HF, HD, HA, HB, and HC treatments. Group 2 can be further subdivided into three subgroups: one subgroup containing HD and HF, another containing HB and HC, and a third subgroup consisting solely of the HA treatment.

### 3.8. Coupling Relationship Between Vegetation Community Characteristics, Soil Physical and Chemical Properties, and Soil Fungal Community

**Figure 12:**
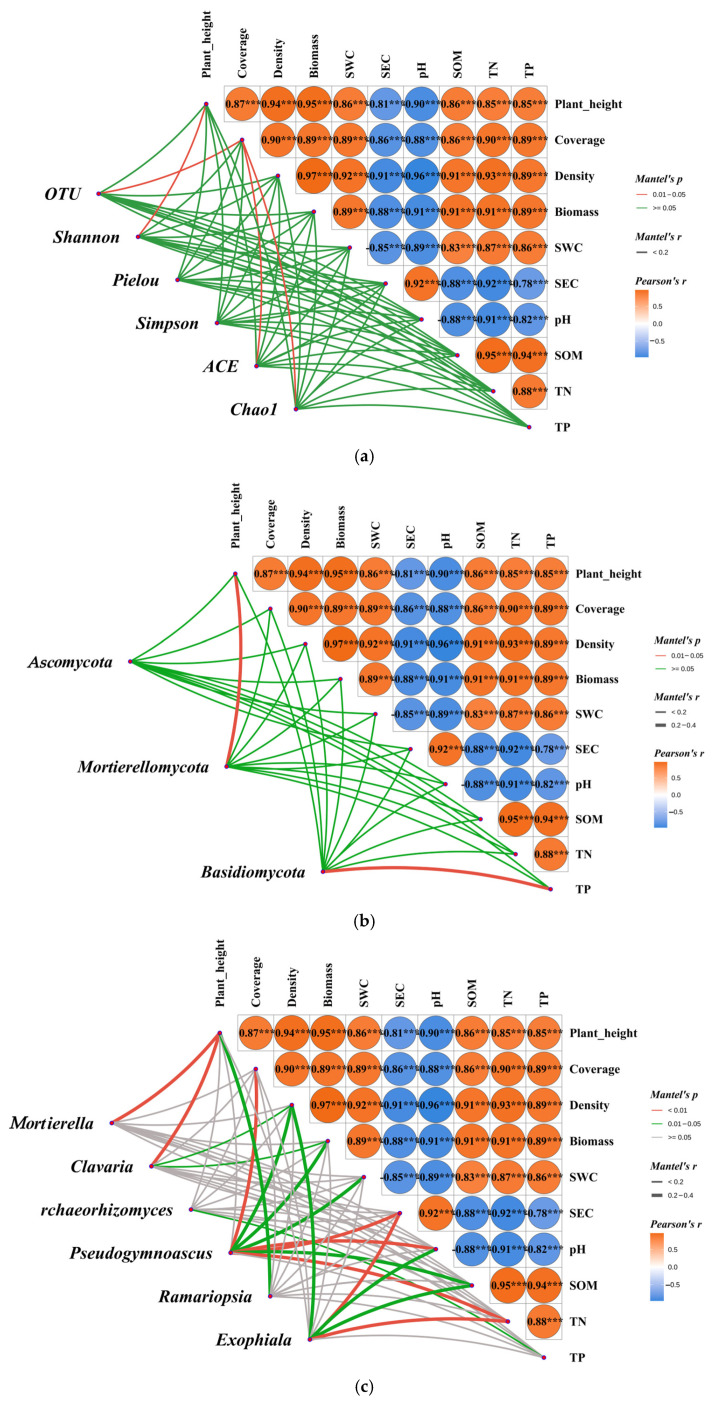
Mantal analysis of vegetation community characteristics, soil physical and chemical properties, and soil fungal community. Note: *** denotes *p* < 0.001. (**a**) α diversity, (**b**) bacterial phylum level, and (**c**) bacterial genus level.

Mantel analysis revealed that there was a large number of extremely significant correlations between the four plant community indices and six soil physicochemical indices (Figure 12). Specifically, SEC and pH exhibited an extremely significant positive correlation (*p* < 0.001), and both SEC and pH showed extremely significant negative correlations with the other four plant indices and four soil physicochemical indices (*p* < 0.001).

Furthermore, significant correlations exist between the aforementioned ten plant and soil indicators and the soil fungal community structure (α diversity, phylum-level abundance, and genus-level abundance) (Figure 12). At the α diversity level (Figure 12a), the number of soil fungal community OTUs exhibited a significant correlation with vegetation coverage (*p* < 0.05). The Shannon index exhibited a significant correlation with vegetation height (*p* < 0.05); both the Ace index and Chao1 index showed significant correlations with vegetation coverage (*p* < 0.05); while the Pielou index and Simpson index demonstrated no significant correlation with any of the 10 vegetation and soil indices. At the phylum level (Figure 12b), the abundance of Mortierellomycota was significantly correlated with vegetation height (*p* < 0.05). The abundance of Basidiomycota was significantly correlated with TP (*p* < 0.05). Ascomycota did not show any significant correlation with any soil physicochemical properties or plant community characteristics. At the genus level (Figure 12c), the abundance of *Mortierella* was highly significantly correlated with plant height (*p* < 0.01).The abundance of *Clavaria* was highly significantly correlated with plant height (*p* < 0.01) and significantly correlated with density and biomass (*p* < 0.05). The abundance of *Archaeorhizomyces* was significantly correlated with TP (*p* < 0.05). The abundance of *Pseudogymnoascus* was highly significantly correlated with coverage, SEC, pH, and TN (*p* < 0.01), and significantly correlated with density, biomass, soil water content (SWC), and SOM (*p* < 0.05). The abundance of *Ramariopsia* was significantly correlated with plant height (*p* < 0.05). The abundance of *Exophiala* was highly significantly correlated with SEC and TN (*p* < 0.01), and significantly correlated with density, pH, and SOM (*p* < 0.05).

**Figure 13:**
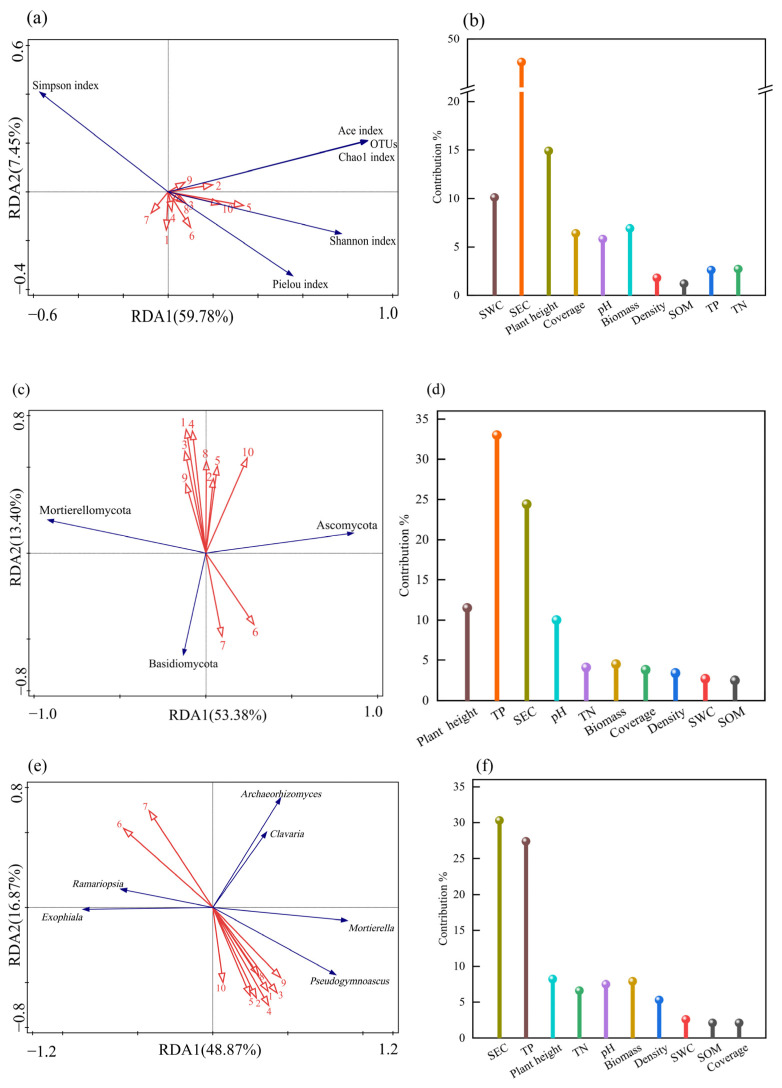
RDA of plant community characteristics, soil physical and chemical properties, and soil fungal community structure. Note: red arrows indicate plant community characteristics and soil factors, and blue arrows indicate the fungal community structure. (**a**,**b**) α diversity; (**c**,**d**) bacterial phylum level; (**e**,**f**) bacterial genus level. 1: Plant height; 2: Coverage; 3: Density; 4: Biomass; 5: SWC; 6: SEC; 7: pH; 8: SOM; 9: TN; 10: TP.

Redundancy analysis (RDA) revealed that the explained variances of soil fungal community α diversity, plant community characteristics and soil physicochemical properties along Axes I and II were 59.78% and 7.45%, respectively, with a cumulative explained variance of 67.23% (Figure 13a,b). Soil electrical conductivity emerged as the primary driving factor, with a contribution rate of 47.60%; its spatial heterogeneity significantly influenced fungal community composition (*p* < 0.05). Vegetation height maintained a secondary core regulatory role, with a contribution rate of 14.90%, which proves the continuous effect of plant vertical structure on microbial habitat screening. The Simpson index exhibited negative correlations with all ten vegetation–soil indices, while the other five soil fungal diversity indices showed positive correlations with most vegetation–soil indices.

The explanatory rates of the phylum-level abundance of soil fungal communities for plant community characteristics and soil physicochemical properties on the first and second principal axes were 53.38% and 13.40%, respectively, with a cumulative explanatory rate of 66.78% (Figure 13c,d). TP, as the primary driving factor, contributed 33.00% to the variation, and its spatial heterogeneity significantly influenced the phylum-level composition of fungal communities (*p* < 0.05). SEC played a secondary regulatory role with a contribution rate of 24.40%, demonstrating that soil physicochemical properties have a significant impact on the phylum-level composition of fungal communities. The abundance of Basidiomycota showed a positive correlation with pH and SEC, while exhibiting negative correlations with the other eight soil–vegetation indicators.

The explanatory rates of the genus-level abundance of soil fungal communities for plant community characteristics and soil physicochemical properties on the first and second principal axes were 48.87% and 16.87%, respectively, with a cumulative explanatory rate of 65.74% (Figure 13e,f). SEC, as the primary driving factor, contributed 30.30% to the variation, and its spatial heterogeneity significantly influenced the genus-level composition of fungal communities (*p* < 0.05). TP maintained a secondary regulatory role with a contribution rate of 27.40%, demonstrating that soil physicochemical properties have a significant impact on the genus-level composition of fungal communities. The abundances of *Mortierella* and *Pseudogymnoascus* showed negative correlations with pH and SEC, while exhibiting positive correlations with the other eight soil–vegetation indicators.

## 4. Discussion

### 4.1. Effects of Poaceae Mixture Sowing on Community Characteristics of Artificial Grassland and Soil Physicochemical Properties

As a key technology for establishing artificial grasslands, the Poaceae mixture technology achieves efficient utilization of environmental resources by optimizing the spatial arrangement of forage species, thereby enhancing grassland productivity and stability [8,16,17]. Based on survey of a 22-year Poaceae mixture grassland on the Qinghai–Tibet Plateau, this study found that mixed sowing has a significant impact on the biomass of vegetation communities in alpine regions. This phenomenon may stem from aboveground–belowground synergistic ecological processes: aboveground, canopy separation enables efficient utilization of light resources; belowground, vertical root stratification promotes the formation of soil macroaggregates, enhancing the rate of organic matter accumulation. Ultimately, biomass is significantly increased through niche complementarity [18,19]. The regulatory effect of the complexity of mixed-sown grass species combinations on soil physicochemical properties is equally significant. The increase in soil moisture content in mixed-sown grasslands can be attributed to vertical root stratification, where deep roots absorb water from deeper layers while shallow roots reduce surface runoff, creating a vertical gradient of water utilization [20]. The enhancement of soil organic matter content in mixed-sown grasslands originates from the synergistic interaction between plant roots and microorganisms. The penetration of coarse roots of *Elymus nutans* promotes the formation of soil macroaggregates and reduces organic matter mineralization. The mucilage secreted by the fine roots of *Poa crymophila* cv. Qinghai, together with the malic acid released by the fibrous roots of *Festuca sinensis* cv. Qinghai, jointly activates rhizosphere fungi, thereby enhancing the efficiency of organic matter accumulation by encapsulating microbial residue carbon [21].

Among the different Poaceae mixture sowing treatments, the HC treatment demonstrated the optimal performance in terms of community density and biomass. Simultaneously, this treatment significantly decreased SEC and pH value, while enhancing the contents of SOM and TN. The HD treatment ranked second only to the HC treatment in terms of community density and biomass, and it exhibited the highest vegetation plant height. The above results indicate that in Poaceae mixture sowing treatments involving three or four Poaceae species, with *Elymus nutans* serving as the dominant grass, a relatively high canopy structure is established; *Poa crymophila* cv. Qinghai and Festuca sinensis cv. Qinghai filled the gaps in vertical and horizontal space through their strong tillering capacity and differences in root distribution, respectively. Through niche complementarity, these species significantly improved resource utilization efficiency, thereby increasing the density and biomass of the grassland community [22]. This finding is consistent with the research conclusions of Xie et al. [23]. and Liu et al. [24].

Monoculture of *Elymus nutans* (HA) performed the worst among all treatments, confirming the ecological vulnerability of single-species communities. This finding aligns with the research conclusions of Ma et al. [25] and Shi et al. [17]. Although *Elymus nutans* is a dominant species in alpine regions, its monoculture tends to lead to a singularization of resource utilization and a decline in the community’s resistance to disturbances [17]. The adaptability and productivity of the community were significantly enhanced through mixed sowing, which introduced functionally complementary species like *Poa crymophila*, (cold-tolerant and trampling-resistant) and *Festuca sinensis* (resistant to barrenness) [25,26]. However, the community characteristics and soil improvement effects of the HF treatment (with the highest number of grass species) did not significantly outperform those of the HA treatment; in fact, some indicators even declined. The reason lies in the “Matthew effect” triggered by an excessive number of grass species in the HF treatment within the alpine habitat of the Qinghai–Tibet Plateau. Although the dominant species, *Elymus nutans*, constructs a high canopy to intercept light resources, the subdominant species, *Poa poophagorum*, experiences intensified light competition due to ecological niche overlap, resulting in reduced photosynthetic efficiency. Simultaneously, competition for water and nutrients between deep-rooted *Poa crymophila* and shallow-rooted *Festuca sinensis* disrupts root exudate interactions, suppresses rhizosphere nitrogen-fixing bacteria activity, and leads to imbalanced resource allocation. These competitive exclusions ultimately impair the stable supply capacity of the HF treatment [17,24,27]. In summary, mixed sowing should adhere to the principle of “moderate diversity”—that is, an excessive number of grass species is not necessarily beneficial. Instead, optimal combinations should be selected through niche differentiation testing to avoid negative effects caused by resource competition.

### 4.2. Effects of Poaceae Mixture Sowing on Soil Fungal Community Structure and Functional Changes

The number of OTUs and diversity of soil fungal communities are important indicators reflecting the health of soil and vegetation. Different grass species directly influence the number of fungal community OTUs and their diversity due to variations in root exudates and litter [28]. This study found that the soil fungal OTU count and Shannon diversity index were significantly higher in the Poaceae mixture sowing HD treatment compared to the monoculture HA treatment, which is consistent with the research findings of Zhao et al. [29] in the Qilian Mountains region. The underlying mechanisms stem from three aspects: First, the roots of mixed-sown grass species secrete secondary metabolites such as malic acid and phenolic acids, attracting the colonization of different functional groups including arbuscular mycorrhizal fungi and saprophytic fungi, thereby increasing the richness of fungal taxa [21,30]. Second, the vertical distribution of roots forms a soil pore gradient, facilitating the extension of fungal hyphae among aggregates. Additionally, the synergistic action of deep and shallow roots reduces surface runoff and maintains soil moisture, creating microhabitats that allow the coexistence of moisture-loving and drought-tolerant fungi [18,19]. Third, the differential utilization of light, water, and nutrients among grass species reduces the intensity of resource competition, avoiding the homogenization of fungal communities caused by resource concentration in monoculture systems, and promoting a shift from competition to coexistence within the fungal community [22,31]. The above research findings confirm that mixed sowing with moderate species richness optimizes soil nutrients and the plant growth environment through niche complementarity, creates diversified fungal habitats, and effectively enhances the number of soil fungal operational taxonomic units (OTUs) and α diversity.

In the mixed-sown grasslands of Poaceae forage grass on the Qinghai–Tibet Plateau, the dominant fungal phyla at the phylum level were all Ascomycota, Mortierellomycota, and Basidiomycota. This is generally consistent with the research findings of Yu et al. [32], indicating that these phyla are the most abundant and play important roles in the soil fungal communities of mixed-sown Poaceae grasslands on the Qinghai–Tibet Plateau. Meanwhile, the study found that different mixed-sown grass species could significantly alter the relative abundance of each fungal phylum in soil fungi. The abundance of Ascomycota in HD and HF treatments was significantly higher than that in HA treatment, while the abundance of Mortierellomycota was lower than that in HA treatment. This change may be attributed to the promotion of plant productivity by mixed sowing of Poaceae forage grass, which subsequently increased litter quantity in the grassland and facilitated the proliferation of Ascomycota with lignin-degrading capability. Additionally, mixed-sown grasslands of Poaceae forage grass exhibit well-developed root systems, whose phenolic acid secretions inhibit the growth of Mortierellomycota [29,33]. At the genus level, *Mortierella* emerged as a dominant functional genus in the soil fungal community across all treatments. The abundance of *Mortierella* showed a significant positive correlation with grassland community productivity, a finding that confirms its pivotal role as a plant growth-promoting fungus in nutrient cycling [33]. Notably, the genus *Romagnesiella* was only detected in the HE treatment (9.43%), belonging to the phylum *Ascomycota*, and is commonly found in specific plant rhizospheres or areas rich in organic matter. The emergence of this phenomenon may be attributed to the selective effect of specific root exudates from *Festuca kryloviana* cv. Huanhu. This finding provides new insights into the theory of plant–microbe-specific interactions [34].

Single-factor molecular network analysis revealed that Poaceae mixture sowing treatments significantly enhanced the complexity of soil fungal community interaction networks: the number of network edges in Poaceae mixture sowing treatments HB (401 edges), HE (490 edges), and HF (494 edges) were all significantly higher than that in the monoculture HA treatment (368 edges). This finding aligns with the conclusion proposed by Guo et al. [35] that there is a positive correlation between plant community complexity and microbial network complexity. Meanwhile, this study demonstrated that the increase in the number of edges was not driven by changes in node quantity but rather stemmed from the reconfiguration of microbial interaction relationships. Poaceae mixture sowing treatments promoted interactions among functional microbial groups by increasing litter input and root exudates, thereby creating differentiated soil microenvironments [36]. Across all experimental treatments, the proportion of positively correlated edges exceeded 50%. Notably, in the monoculture HA treatment, resource limitations compelled microorganisms to strengthen positive interactions to maintain functional stability—a phenomenon similar to the findings reported by Yang et al. [37]. The HA treatment was dominated by Entorrhizomycota and Chytridiomycota as the core phyla, whereas Poaceae mixture sowing treatments shifted toward dominance by Mortierellomycota (exhibiting plant growth-promoting traits such as ACC deaminase secretion) and Ascomycota (possessing lignin degradation capabilities). The ecological mechanism underlying this shift in dominant phyla lies in the fact that Poaceae mixture sowing promotes synergistic interactions among functional microbial groups by optimizing resource allocation and enhancing microhabitat heterogeneity. Mortierellomycota enhances host stress resistance by secreting plant growth-regulating substances, while Ascomycota improves nutrient cycling efficiency through the degradation of complex organic matter, ultimately forming a more stable soil–microbe–plant interaction network [38].

FUNGuild functional analysis revealed that Poaceae mixture sowing significantly reshaped the functional structure of the fungal community: compared to the monoculture HA treatment, the functional abundance of plant pathogen was significantly reduced in the Poaceae mixture sowing HF treatment. This “disease-suppressing and growth-promoting” effect is consistent with the research findings of Gao et al. [39]. The ecological mechanism lies in the fact that the healthy soil environment and diversified plant community formed by Poaceae mixture sowing can promote the secretion of antibiotic substances by beneficial microbial groups, directly inhibiting the reproduction of pathogens [40]. Mixed sowing of 3–4 forage grass species optimizes the soil microenvironment through niche complementarity, providing suitable habitats for functional microbial groups and demonstrating superior performance in maintaining both α diversity and functional balance of the fungal community [21,23,36]. It is noteworthy that approximately 30% of fungal functions remain undefined, and their metabolic potential and ecological roles require further exploration. Future research could integrate metagenomic technologies to deeply dissect the metabolic pathways and ecological functions of key microbial groups, providing more precise microbiological evidence for the ecological restoration of alpine grasslands.

### 4.3. Correlation Analysis Between Soil Physical and Chemical Properties, Plant Community Characteristics, and Soil Fungal Community Diversity

Based on Mantel analysis, this study elucidated the interaction patterns within the plant–soil–microbe system in a Poaceae mixture grassland. The results revealed a significant coupling relationship between soil physicochemical properties and plant community characteristics. Specifically, soil pH exhibited a highly significant positive correlation with SEC, while both factors demonstrated highly significant negative correlations with other soil physicochemical properties and vegetation community characteristics. These findings are consistent with the research results of Han et al. [41], Feng et al. [42], and Gao et al. [14], indicating that elevated soil nutrient content can promote the growth of plant communities, whereas saline–alkali conditions inhibit plant growth. Further analysis revealed significant correlations between fungal community α diversity, phylum/genus-level abundances, and plant–soil characteristics. Specifically, the Shannon index, Mortierellomycota abundance, and *Mortierella* abundance all exhibited significant correlations with plant height. These findings suggest that mixed-sown Poaceae grasslands can enhance soil fertility improvement and promote fungal community optimization through “plant–soi–microbe” interactions, thereby establishing a sustainable ecological cycle [29,43].

Redundancy analysis revealed that SEC is a key regulatory factor driving changes in fungal community diversity and structure in mixed-sown Poaceae grasslands. SEC regulates the microbial community in mixed-sown Poaceae grasslands through three primary mechanisms: (1) High SEC alters soil osmotic pressure, disrupting fungal cellular water balance and restricting the growth of certain sensitive microbial groups [44]. (2) Elevated SEC may coincide with sodium ion accumulation, modifying nutrient availability and affecting fungal decomposition efficiency of organic matter [14]. (3) Salt stress intensifies resource competition among fungi, such as competition for carbon sources between saprotrophs and pathogens, leading to the restructuring of community functional composition [45]. This process indicates that SEC not only directly influences the soil microbial community in mixed-sown grasslands by affecting soil osmotic pressure and soil fertility but also indirectly regulates the stability of the microbial community through resource competition [14,44,45].

Future research will prioritize the supplementation of relevant data on untreated degraded soil to establish a comprehensive effect evaluation framework encompassing ‘natural degradation–monoculture restoration–mixed-sowing restoration’. This will enable a more rigorous analysis of the impacts of sowing treatments on vegetation, soil physicochemical properties, and soil microorganisms, thereby providing stronger scientific support for the current comparative conclusions between monoculture and mixed-sowing treatments. Meanwhile, it is essential to integrate metagenomics to deepen the theoretical understanding of plant–soil–microorganism interactions, further offering a scientific basis for ecological restoration.

## 5. Conclusions

This study demonstrates that mixed sowing of Poaceae forage grasses with moderate species richness (3–4 species)—such as the HC treatment (*Elymus nutans* Griseb. + *Poa crymophila* cv. Qinghai + *Festuca sinensis* cv. Qinghai) and the HD treatment (*Elymus nutans* Griseb. + *Poa crymophila* cv. Qinghai + *Festuca sinensis* cv. Qinghai + *Poa poophagorum*)—can significantly promote plant growth (by increasing density and biomass), improve soil properties, and reshape the soil fungal community structure (increasing the abundance of Ascomycota and the number of operational taxonomic units, along with α diversity indices such as the Shannon index and Pielou index, accompanied by a rise in beneficial fungal groups and a decline in pathogens). Environmental driver analysis revealed that soil electrical conductivity and total phosphorus content are the core factors influencing fungal community structural changes (α diversity, as well as abundance of the phylum and genus levels). Therefore, we recommend adopting the HC or HD mixed-sowing strategy for alpine grassland restoration, while simultaneously regulating soil electrical conductivity and total phosphorus to optimize the recovery effects on the fungal community.

## Figures and Tables

**Table 1:** Changes in grassland vegetation height, cover, density, and aboveground biomass under different treatments.

Year	Treatment	Plant Height/cm	Plant Coverage/%	Density /(Plant·m^−2^)	Aboveground Biomass/(g·m^−2^)
2023	HA	35.79 ± 1.17 c	72.00 ± 2.65 d	708.00 ± 18.68 d	951.00 ± 19.47 c
	HB	40.54 ± 1.40 b	80.00 ± 4.36 bc	791.00 ± 11.79 c	1241.33 ± 77.09 b
	HC	42.12 ± 0.97 b	90.33 ± 3.51 a	913.33 ± 25.54 a	1580.00 ± 141.27 a
	HD	45.86 ± 2.15 a	92.33 ± 3.21 a	855.00 ± 16.37 b	1574.00 ± 94.78 a
	HE	39.23 ± 0.83 b	84.00 ± 3.61 b	734.00 ± 21.66 d	1154.67 ± 97.39 bc
	HF	32.51 ± 2.21 d	76.33 ± 3.21 cd	651.33 ± 18.82 e	978.67 ± 97.96 c
2024	HA	32.00 ± 2.65 c	75.33 ± 3.79 d	665.33 ± 20.65 d	1079.67 ± 131.25 c
	HB	39.33 ± 2.08 b	86.33 ± 3.21 bc	800.67 ± 15.37 c	1388.00 ± 95.39 b
	HC	50.00 ± 2.65 a	95.33 ± 2.08 a	942.33 ± 17.93 a	1645.00 ± 76.92 a
	HD	45.00 ± 4.58 a	90.67 ± 3.21 ab	902.00 ± 18.52 b	1431.00 ± 64.37 b
	HE	34.00 ± 3.61 bc	83.00 ± 4.36 c	811.33 ± 20.53 c	1206.67 ± 91.95 c
	HF	28.33 ± 2.08 c	77.00 ± 2.00 d	692.67 ± 15.50 d	1022.33 ± 124.23 c

Note: Data are presented as mean ± standard deviation; different lowercase letters within the same column indicate significant differences between treatments at the *p* < 0.05 level.

## Data Availability

The original contributions presented in this study are included in the article; further inquiries can be directed to the corresponding author.
